# Acute Kidney Injury: Definition, Management, and Promising Therapeutic Target

**DOI:** 10.7759/cureus.51228

**Published:** 2023-12-28

**Authors:** Meaad A Almazmomi, Ahmed Esmat, Anjum Naeem

**Affiliations:** 1 Pharmaceutical Care Department, Ministry of National Guard - Health Affairs, Jeddah, SAU; 2 Pharmacology Department, Faculty of Medicine, King Abdulaziz University, Jeddah, SAU

**Keywords:** therapeutic, tlr-4, cytokine storm, inflammation, acute kidney injury

## Abstract

Acute kidney injury (AKI) is caused by a sudden loss of renal function, resulting in the build-up of waste products and a significant increase in mortality and morbidity. It is commonly diagnosed in critically ill patients, with its occurrence estimated at up to 50% in patients hospitalized in the intensive critical unit. Despite ongoing efforts, the death rate associated with AKI has remained high over the past half-century. Thus, it is critical to investigate novel therapy options for preventing the epidemic. Many studies have found that inflammation and Toll-like receptor-4 (TLR-4) activation have a significant role in the pathogenesis of AKI. Noteworthy, challenges in the search for efficient pharmacological therapy for AKI have arisen due to the multifaceted origin and complexity of the clinical history of people with the disease. This article focuses on kidney injury's epidemiology, risk factors, and pathophysiological processes. Specifically, it focuses on the role of TLRs especially type 4 in disease development.

## Introduction and background

Acute kidney injury (AKI) is a common and serious clinical illness defined by a substantial decrease in kidney function within hours to days and is often reversible [1]. AKI is a spectrum of morbidity ranging from subclinical damage, with a minimal, rising serum creatinine (SCr) level, to anuric renal failure, which significantly influences healthcare costs [2]. It is linked to cardiovascular disease (CVD), end-stage renal disease (ESRD), hypertension, and death [3-5]. Renal function may not improve or only partially recover in severe AKI needing dialysis [6]. However, it has become more evident that AKI patients who seem to have fully recovered still run the risk of long-term renal problems [7-9]. AKI survivors are more likely to have chronic kidney disease (CKD) and ESRD, which have severe economic, social, and human costs [10,11]. AKI affects all ages in the community and hospital. It is a major reason for worldwide morbidity and death [2]. In the community, AKI is predicted to affect 20-200 people out of every million people [12]. It is associated with hospital mortality rates exceeding 50% in severe cases [13-15]. Since AKI is becoming severe, two million people worldwide are estimated to die from it yearly [10,15]. AKI prevalence varies between high-income and low-to-middle-income nations. The prevalence of AKI is lower in high-income nations than in low-to-middle-income countries, where endemic illnesses like malaria and tainted water are major contributors to the high burden of AKI [16].

Even though the pathogenesis of AKI is still not well recognized, it is often a complex condition regularly implicated by hemodynamic instability, sepsis, and medication toxicity. Regardless of the specific cause, some distinct pathophysiologic processes, including endothelial dysfunction, oxidative stress (OS), changes in microcirculation, and intrarenal inflammation, occur concurrently and in order [17-19]. As systemic and intrarenal inflammation are linked to AKI, developing the knowledge of the molecular and cellular processes underlying the inflammatory response has great potential for developing AKI prevention or treatment strategies [20]. The activation of Toll-like receptor-4 (TLR-4) leads to the production of cytokines and chemokines and renal leucocyte infiltration. Additionally, it alters renal metabolism and circulation and causes endothelial and tubular dysfunction [21]. There is evidence of an inflammatory response mediated by TLR-4 to have a substantial part in the pathophysiology of AKI. In order to avoid renal inflammation and eventual kidney damage, inhibiting TLR-4 and its downstream effectors may be a useful therapeutic approach [22].

## Review

Kidney anatomy and physiology

Kidneys are supported by adipose tissue and have a fibrous outer covering called the renal capsule. The kidney's outer layer, the renal cortex, and the renal medullam which its inner layer, are two distinct anatomical structures. The renal medulla contains the renal pyramids and the renal columns. Renal pyramids may be recognized by their triangular form; renal columns are arranged inside them. Lobes of the kidney are made up of renal pyramids and renal columns. The renal pyramids serve as the starting point for the minor calyces, which then converge to form the major calyces. The final destination for the major calyces is the renal pelvis, which collects the urine made by the kidney. The urine leaves the renal pelvis through the ureters and is kept in the bladder until excretion [23].

The kidney’s functional components, called nephrons, are little blood-filtering organelles found in the cortex and the medulla. Nephrons stretch from the renal capsule to the cup-shaped renal pelvis through the cortex and medulla. A single kidney has more than a million nephrons, around 2-4 cm long [23]. The kidneys receive about 20% of the heart’s output. Blood flow is selectively distributed throughout the kidneys. Renal arteries branch out into glomerular afferent arterioles, creating a capillary network accountable for glomerular filtration [17].

Oxidative metabolism is the main way that adenosine triphosphate (ATP) is produced in the kidney. Over 97% of ATP synthesis inthe cortex originates from oxidative metabolism, whereas up to 33% of energy in the medulla comes from glycolysis [24]. The kidneys are complicated organs responsible for many tasks and chemicals essential for maintaining homeostasis [25]. The kidneys' basic functions are eliminating metabolic wastes, maintaining fluid and electrolyte balance, and contributing to acid-base equilibrium. Kidney hormones have a significant role in blood pressure regulation, red blood cell formation, and bone metabolism [26-31]. The kidneys are essential in maintaining the intracellular and extracellular conditions for all cells to function optimally [32].

Definition and staging criteria of AKI

AKI results from a rapid and, in many cases, a temporary decline in kidney function. Although blood urea nitrogen (BUN) or creatinine values may be within normal range right after a renal injury, a decrease in urine production may be the only indication of AKI [33]. An abnormal and rapid increase in SCr levels or a decrease in urine production characterizes this disorder, leading to a build-up of water, sodium, and waste products such as creatinine, urea, and uric acid. An increase in morbidity and death strongly correlates with it [34]. Acute renal injury is described as having a duration of no more than seven days and is accompanied by signs of functional or structural impairment [1]. As a result of the inefficiency of its treatment, this condition often necessitates a prolonged stay in the hospital, which drives up healthcare costs [34,35].

The latest classification of AKI was implemented following the Kidney Disease Improving Global Outcomes Guidelines (KDIGO) 2012, which come from both the Acute Kidney Injury Network (AKIN) criteria and the Risk, Injury, and Failure, Loss, and End-stage kidney disease (RIFLE) criteria. A diagnosis of AKI might be made in any one of the following clinical scenarios. A 48-hour period during which SCr levels increase by 0.3 mg/dL or higher; SCr levels that have increased by 1.5 times or more from baseline in the last week; or a urine flow rate for at least six hours of less than 0.5 mL/kg/h [36,37]. Moreover, KDIGO suggests categorizing the severity of AKI into stages. It depends on the SCr increase or drops in urine output. The first stage is characterized by a decrease in urine output of 0.5 mL/kg/h lasting more than 6 hours or an increase in SCr of 1.5-1.9 times over baseline. Stage two is a decrease of 0.5 mL/kg/h in urine output over 12 hours or more or a 2.0-2.9-fold increase in Scr from baseline. Stage 3 is defined as a decrease in urine output to less than 0.3 mL/kg/h for 24 hours or more, as well as a tripling of SCr, or anuria lasting 12 hours or more (Table 1) [38].

**Table 1:** AKI staging proposed by KDIGO This table is adapted from [38] KDIGO - Kidney Disease Improving Global Outcomes Guidelines

Stage	Serum creatinine	Urine output
1	1.5–1.9-fold baseline or an increase by ≥0.3 mg/dL (≥26.5 µmol/L)	<0.5 mL/kg/h for 6–12 h
2	2.0–2.9-fold baseline	<0.5 mL/kg/h for ≥12 h
3	3-fold baseline or an increase in serum creatinine up to or greater than 4mg/dl or an acute rise by at least 5mg/dl or beginning of renal replacement therapy or a drop in eGFR <35 mL/min/1.73 m^2 ^for patients <18 years	<0.3 mL/kg/h for ≥24 h or anuria ≥12 h

Epidemiology of AKI

The prevalence of AKI, the rise in incidence, and the severe morbidity and mortality have been demonstrated in several studies [39-42]. AKI is thought to impact more than 13 million individuals annually worldwide, with significant regional differences based on various nations, regions, and economics [13,43]. The prevalence of AKI varies widely between studies, with some reporting a 3.2%-20% incidence among hospitalized patients and others reporting a prevalence as high as 67% among the intensive critical unit (ICU) patients [40,44-47]. Inconsistent application of the established AKI categorization criteria and population differences can account for these variations [16].

Using KDIGO-equivalent criteria, published meta-analyses of the worldwide burden of AKI found that the pooled incidence of AKI in the hospitalized population was 19.4% in Eastern Asia, 7.5% in Southern Asia, 31.0% in Southeastern Asia, 9.0% in Central Asia, and 16.7% in Western Asia [13,43]. The estimated pooled AKI-associated death rates are still high, which was 36.9% in Eastern Asia, 13.8% in Southern Asia, 23.6% in Western Asia in adults, and 14% in children [13]. These staggering rates of AKI among hospitalized patients show that Asia, like the rest of the world, is facing a significant medical burden due to AKI. However, because of the small number of papers available for inclusion in the meta-analysis, it remains challenging to establish the true prevalence of AKI in Asia [48]. In a retrospective cohort study in King Abdulaziz University Hospital (KAUH), Jeddah, Saudi Arabia, the incidence of AKI was 39% of all adults admitted to the ICU. Results showed that sepsis (44.5%), Coronavirus disease 2019 (COVID-19) (31.2%), and hypotension (23.5%) were the most common underlying medical condition associated with AKI [49]. According to the KDIGO definition of AKI, 37.4% of critically ill pediatric patients were found to have AKI in a multi-center prospective cohort research conducted in the Kingdom of Saudi Arabia [50].

Risk factors for AKI

Considering the fact that AKI is linked to a large increase in mortality and morbidity. Identifying high-risk populations and clinical diseases, as well as high-risk circumstances and procedures, is a significant step in avoiding AKI. This step allows for emerging strategies to reduce AKI's frequency and severity. Hospitalized patients frequently exhibit a high frequency of AKI risk factors, with high incidence in particularly ICU patients [51]. According to the findings of the vast majority of studies, advancing age is an independent risk factor that may have a role in the development of AKI. In 2009, the United States Renal Data System (USRDS) reported incidence rates of AKI in the United States using three different datasets that covered the period 1995 to 2007. They discovered that the elderly population with age greater than 85 years old had the highest rate of AKI incidence progression [52]. Age may be an independent risk factor for many different causes, including diminished residual renal function, the existence of comorbidities, and increased susceptibility to infections [53,54]. The patient's risk of developing AKI is increased by the presence of obesity, male sex, and genetic factors [55-57].

Comorbid conditions are important risk factors associated with AKI. Comorbidities that increase the risk of the development of AKI include diabetes, heart failure, hyperuricemia, and hypoalbuminemia [55-60]. In addition, it has been reported that rhabdomyolysis, surgical procedures, and mechanical ventilation are all related to an increased risk of AKI [61]. Chronic renal disease or proteinuria before the event seems to be a substantial risk factor [55].

Nephrotoxic drugs were identified as those with the potential to negatively impact renal function, either directly or via impairing renal perfusion, while acknowledging that the severity of their toxicity may vary depending on the clinical situation [62]. AKI risk can also be raised by using multiple nephrotoxic medicines together. Some examples of the most common nephrotoxic agents are non-steroidal anti-inflammatory drugs (NSAIDs), aminoglycosides, angiotensin-converting enzyme inhibitors (ACEIs), angiotensin receptor blockers (ARBs), amphotericin B, calcineurin inhibitors, chemotherapeutic agents such as cisplatin and ifosfamide, radiocontrast agents and diuretics [51,62-64].

In addition, some conditions, such as hypotension, hypovolemia, and exposure to iodinated contrast media, play a part in developing AKI. AKI is frequently linked to sepsis and severe infections, particularly when a surgical procedure is involved [62].

Classification and etiology of AKI

The pressure difference between the glomerulus and the Bowman space drives glomerular filtration. This pressure gradient is influenced by blood flow through the kidneys and is directly regulated by the combined resistances of afferent and efferent vascular pathways. However, renal blood flow reduction is a frequent pathologic mechanism for decreasing glomerular filtration rate (GFR), regardless of the cause of AKI [65,66]. AKI's underlying causes can be prerenal, intrinsic, or postrenal. Numerous diverse causes fall under each of these categories. The first step in treating the patient is understanding the cause [67].

Pre-renal

The most frequent cause of AKI is prerenal azotemia, also identified as functional AKI. It is caused by various pathophysiologic events and accounts for up to 50% of all AKI cases [68-70]. Prerenal azotemia is identified by an increase in SCr or a decrease in urine flow without the presence of positive indicators for urinary cell injury [70]. Prerenal AKI may have normal underlying kidney function, but reduced renal perfusion from decreased intravascular volume or reduced effective arterial blood volume causes a lower GFR without affecting the renal parenchyma [71,72]. In order to maintain a normal GFR, adequate renal perfusion is necessary, as is widely known. Since the kidneys receive up to 20% of cardiac output, any systematic decrease in circulating blood volume or a single intrarenal circulation failure might significantly impact renal perfusion [73]. Many conditions can cause hypovolemia, resulting in decreased renal perfusion, which involves hemorrhage, gastrointestinal fluid losses (vomiting, diarrhea), and kidney losses such as over-diuresis and diabetes insipidus. Additionally, anaphylaxis, cirrhosis, nephrotic syndrome, cardiogenic shock, septic shock, and cirrhosis are all pathophysiologic diseases that lower effective circulation volume, resulting in decreased effective arterial blood volume [74]. Aortic and cardiac baroreceptors are activated when effective arterial blood volume is decreased, starting a cascade of humoral and neurological reactions to limit any decline in renal blood flow and GFR [70].

Several medications have been linked to the prerenal cause of AKI. It should be noted that ACEIs and ARBs can affect renal perfusion by producing vasodilation of the efferent arteriole, lowering intraglomerular pressure, and lowering GFR. The use of NSAIDs hampers the production of vasodilatory prostaglandins. NSAIDs can potentially disrupt the normal ratio of vasodilatory to vasoconstrictive agents found in renal microcirculation. This can lead to a vasoconstriction of the afferent arteriole and a reduction in GFR. Reducing renal function may be connected with using these medicines and others, inhibiting the typical homeostatic reactions to volume deprivation [75]. Since the renal parenchyma is unharmed, pre-renal AKI typically resolves itself rather quickly after restoring renal perfusion. On the other hand, if the hypoperfusion is significant, it could cause ischemia, leading to acute tubular necrosis (ATN) [76].

Intrinsic Renal

Intrinsic kidney damage is the most prevalent form of kidney damage [73]. Because there are many distinct types of kidney injuries, evaluating the patient's condition might be difficult. In most cases, four components of the kidney are implicated: the tubules, the glomeruli, the interstitium, and the intra-renal blood vessels [77]. 

AKI caused by tubule destruction is medically referred to as ATN. ATN is the most common intrinsic kidney injury [77]. The cause is often either nephrotoxic, caused by an endogenous and exogenous toxin that harms the tubular cells, or ischemic due to hypovolemia, extended periods of severe hypotension, or hypoperfusion to the kidneys, such as from hemorrhage, shock, cirrhosis, peritonitis, or infarcts [75,77].

AKI is caused by glomerular injury in severe acute glomerulonephritis (GN) cases. Acute interstitial nephritis (AIN) is a frequent cause of AKI. It accounts for 15%-27% of renal biopsies performed because of this condition [78]. It arises from an allergic response to various drugs, including NSAIDs, beta-lactam antibiotics, penicillin, proton pump inhibitors (PPIs), or infections [73]. Extra-renal antigens produced by medications or infectious agents are likely to cause most AIN cases. These extra-renal antigens may cause AIN by attaching to kidney structures, altering the immunogenetics of native renal proteins, simulating renal antigens, or precipitating as immune complexes, where they act as the site of an immune response or cellular damage [79]. Injury to the intrarenal vessels, which leads to a reduction in renal perfusion and a decrease in GFR, is the cause of vascular damage-related AKI [73].

Post-renal

It develops when there is a sudden blockage of the urine flow, which leads to a rise in the pressure of intra-tubules [80]. The most common causes include calculi in the kidneys or ureters, tumors, blood clots, or any obstruction in the urethra [33]. Acute urinary tract obstruction can result in decreased renal blood flow and inflammatory processes, all of which contribute to decreased GFR [81]. AKI is frequently caused by obstructive uropathy, which accounts for up to 10% of all AKI cases in the general population and an even larger percentage in the elderly [82,83]. Post-renal AKI can develop when there is a blockage at any level of the urine collecting system, such as an obstruction in the ureter, the bladder, or the urethra. If the obstruction is above the bladder, it must involve both kidneys or just one kidney if the patient only has one kidney functioning properly to produce significant renal failure. However, a single kidney obstruction can cause AKI in patients with renal insufficiency. Urinary obstruction can exhibit as anuria or intermittent urine flow, like polyuria followed by oliguria, although it can also show up as nocturia or non-oliguric AKI [73].

Pathogenesis of AKI

The following pathways can be used to explain the pathophysiology of AKI in sepsis. A unified theory explanation of explain the mechanism of sepsis-induced AKI (SI-AKI) have been revealed by Gomez et al., in which they have interacted together as they are the important processes for AKI in sepsis since each of the following roles cannot be identified independently [84].

Changes in Glomerular Hemodynamics

Although during septic AKI, the renal blood flow is enhanced the GFR is not raised but lowered and can stop entirely [85]. Sepsis causes the efferent arteriole to dilate predominately, lowering the hydrostatic pressure in the glomerular capillaries and the net filtration pressure. Because the filtered load of sodium chloride (NaCl) is lower when GFR is lower, energy consumption is also reduced. NaCl Impaired reabsorption by injured tubules increases NaCl transport to macula densa, which is detected by the Na-K-2CL channel and turns off tubuloglomerular feedback (TGF), causing constriction of afferent arteriolar and blunting afferent vasodilatation [86]. Sympathetic nerve activity during sepsis will also counteract afferent vasodilatation and reduce GFR and oliguria [87].

Septic patients with greater central venous pressure (CVP) were more likely to have AKI [88]. In sepsis, high CVP and backward pressure cause renal congestion and increased pressure throughout the renal vascular tree, which impairs renal function [89,90]. Since the renal capsule surrounding the kidney is noncompliant, intrarenal pressure rises exponentially as renal volume increases. This causes a decrease in the trans-renal pressure gradient, which lowers renal blood flow, and an increase in intratubular pressure that offsets net filtration pressure [91]. Most of the data come from patients with cardiovascular disease (CVD), and serious illnesses, where it has been consistently demonstrated that venous congestion contributes to AKI development [92-94].

Microvascular Dysfunction

The equilibrium of oxygen (O2) supply and consumption determines how well renal tissue is oxygenated. ATP generation, the primary energy source used by Na/K-ATPases, which is active pumps in the cell membrane, determines the amount of oxygen required by the kidneys [95]. The kidney O2 supply is regulated properly under steady-state conditions. The generation of mitochondrial ATP, reactive oxygen species (ROS) and nitric oxide (NO), required for homeostatic regulation of renal function depends on adequate O2 supply [96,97]. The kidney is a circulatory organ that receives 25% of cardiac output and has a high energy need with minimal oxygen extraction. The blood flow to the outer medulla is approximately half that to the cortex, resulting in partial oxygen pressures of 10-20 mmHg and 50 mmHg, respectively [98]. Consequently, the outer medulla is a region that is particularly susceptible to circulation problems and hypoxia [99,100].

Decreased capillary density is a hallmark of microvascular dysfunction. Symptoms include increased capillaries receiving inadequate or no blood flow and reduced capillaries receiving adequate blood flow [95]. Sepsis is an example of an inflammatory condition that can significantly affect microvascular function, resulting in a heterogeneous and slow flow. This may cause patchy kidney hypoperfusion and micro-ischemia even without overall hypoperfusion [101,102]. Intact regions with maintained tissue oxygenation may coexist alongside hypoxic zones, and interactions between these regions are thought to be related to enhanced ROS formation [103]. In addition, OS and the resulting synthesis of vasoactive prostaglandins by injured tubular cells further reduce O2 supply by increasing local microvascular occlusion [99,100]. The main result of microvascular injury is decreased peritubular capillary density due to reduced vascular endothelial growth factor (VEGF) and increased transforming growth factor beta (TGF-β) signaling, which leads to hypoxia and renal fibrosis [104].

Endothelial Dysfunction

Endothelium separates extravascular and intravascular spaces. Endothelial cells regulate blood flow, vascular tone, and permeability [105]. They regulate microcirculation and glomerular filtration by generating NO, prostacyclin, and other vasoactive molecules that affect the tone of venules and arterioles and prevent platelet aggregation. The integrity of the endothelium barrier restricts the passage of albumin and bigger endogenous molecules in the urine. The glycocalyx is a network of glycoproteins that lines the extracellular surface of endothelial cells and has an important role in maintaining the integrity of the endothelial barrier. Endothelial dysfunction and glycocalyx damage cause microvascular dysfunction, capillary leakage, and GFR impairment [106,107]. The endothelium undergoes structural alterations after exposure to systemic inflammatory mediators, including the loss of cell-cell contact and glycocalyx breakdown. Activated endothelial cells also up-regulate adhesion molecules, which increases leukocyte and platelet adherence, which affects perfusion, O2 supply, and cell damage and inflammation. Increased vascular permeability and the subsequent development of interstitial edema further impair blood flow, worsening the original injury [99,100].

Injured endothelial cells generate fewer vasodilators (e.g., nitric oxide), which increases the response to vasoconstrictors like endothelin-1, angiotensin II (ANGII), and others, redistributing blood flow. AKI is linked to endothelial vasoconstrictors, vasodilators, and OS imbalance [108]. Inflammatory conditions like sepsis increase NO synthesis globally via inducible nitric oxide synthase (iNOS) production. However, iNOS expression is heterogeneous [109]. Heterogeneous iNOS expression in AKI causes localized NO levels to rise, exacerbated by microcirculatory dysfunction, perpetuating regional oxygen deprivation [84]. Langenberg et al. showed that sepsis increases all iNOS isoforms in the renal cortex but not in the medulla. Thus, iNOS isoform overexpression in the cortex may cause intra-renal shunting [110]. Blood may be diverted from the medulla, causing renal medullar hypoxia [110,111].

Activation of Coagulation Cascade

Systemic thrombin and micro-thrombi development due to abnormalities in coagulation regulatory pathways and inflammatory processes cause organ failure [112]. Sepsis causes coagulation due to vascular damage and inflammation. Immunothrombosis is a tight relationship between inflammation and coagulation: Platelet aggregation releases cytokines and chemokines from platelet granules, recruiting leukocytes and inflaming the area [113]. Antithrombin directly reduces inflammation by boosting prostacyclin synthesis and inhibiting leukocyte recruitment. As a negative acute phase response, activated leukocytes break down and consume numerous natural anticoagulants for increased thrombin generation, or their production is lowered owing to defective synthesis, resulting in a pro-coagulant condition [114].

Oxidative Stress

OS is a disruption in normal molecular and cellular function caused by an imbalance between reactive species production and the cells' natural antioxidant capabilities [115,116]. ROS and reactive nitrogen species cause oxidative stress. ROS are the most significant biological free radicals [117]. Mitochondrion, the body's energy factory, is abundant in the proximal renal tubule, making the renal cortex a major oxygen-using region for energy generation [118]. ROS are primarily produced when cytochrome oxidase reduces oxygen, and they typically originate in the mitochondria during the electron transport chain process [116]. Enzymes from the endoplasmic reticulum and peroxisomes, such as the nicotinamide adenine dinucleotide phosphate hydrogen (NADPH) oxidase complex (NOX), also produce ROS in the kidneys [116,119]. ROS generation is normally balanced by the availability and cellular location of antioxidant enzymes and thiols such as CAT, SOD, GSH and glutathione peroxidase (Gpx) [117,120].

**Figure 1:**
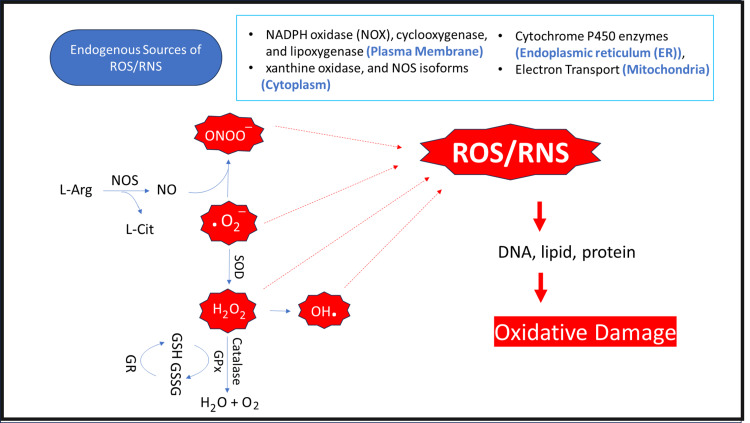
The pathways of reactive oxygen species (ROS) and reactive nitrogen species (RNS) generating Copyright/license: This figure has been modified from an open-source article [123] distributed under the terms and conditions of the Creative Commons CC BY license. ER: endoplasmic reticulum; GPx: glutathione peroxidase; GR: glutathione reductase; GSH: reduced glutathione; GSSG: oxidized glutathione; L-Arg: L-arginine; L-Cit: L-citrulline; NADPH: nicotinamide adenine dinucleotide phosphate hydrogen; NO: nitric oxide; NOS: nitric oxide synthase; O2−: superoxide radical; ONOO−: peroxynitrite; SOD: superoxide dismutase.

About 1%-2% of consumed O2 is transformed into superoxide anions (O2-), the most frequent ROS and a potent precursor of hydrogen peroxide (H2O2) [121,122]. Despite its stability, cellular H2O2 can harm substrates, especially in the presence of the reduced metal ion Fe2+. However, O2- is converted to H2O2 by SOD. H2O2 then transforms into the most reactive and destructive ROS, hydroxyl radical (OH-). Nitric oxide synthase (NOS) converts L-arginine (L-Arg) into NO and L-citrulline (L-Cit). Excess NO competes with SOD and interacts with OH- to create peroxynitrite (ONOO−), which directly damages tubular cells [121, 122]. In healthy cells, mitochondrial or cytosolic CAT or thiol peroxidases catalyze H2O2 into water (H2O) and O2 and are inactivated by antioxidant molecules like GSH (Figure 1) [117,121,123].

ROS are harmful compounds. Even when synthesized during normal respiration, they might cause cumulative damage, eventually leading to cell and tissue dysfunction and disease. Their production rises above natural antioxidant levels with progressive disease and aging [124]. Hypoxic tissues generate excessive ROS during sepsis, increasing GSH consumption [125]. In vivo investigations show that decreasing levels of these endogenous antioxidants cause oxidative damage rather than increased ROS generation [126]. 

High OS contributes significantly to kidney damage through interactions with cellular damage, protein malfunction, and damage to deoxyribonucleic acid (DNA), lipids, and enzymes, which increases mitochondrial permeability [115,116,127,128]. Reduced ATP production and electrochemical gradient are also related to increased mitochondrial permeability, which also causes apoptotic pathways to be activated [129]. ROS directly damages endothelium and cell membranes and glycocalyx, impairing endothelium-dependent vaso-reactivity [130]. They also mediate the vasoconstrictive effect of endothelin-1 and may impair the glomerular hemodynamic [131].

The Role of Renin-Angiotensin-Aldosterone System

Renin-angiotensin-aldosterone system (RAAS) has crucial roles in regulating renal hemodynamics and functioning and in the development of renal disease [132].

Angiotensin I, produced from angiotensinogen (AGT), and then is converted to ANGII in lung endothelial cells by the angiotensin-converting enzyme (ACE) [133]. In addition to the lungs, this mechanism happens in the kidney, heart, and brain vascular beds [134]. Numerous processes in the kidney regulate ANGII production. ANGII concentrations are substantially greater in the renal interstitial fluid than in the circulation [132]. ANGII controls volume and fluid balance through sodium reabsorption and aldosterone production [135]. It also raises blood pressure by constricting systemic vasculature. However, it also impacts arterial pressure through renal hemodynamics, salt and water excretion. The afferent and efferent arterioles constrict, although the efferent arteriole is more affected by ANGII [136]. ANGII works through two functional receptors, angiotensin II type1 receptor (AT1R) and angiotensin II type 2 receptor (AT2R). An increase in local RAAS activity contributes to the development of AKI by increasing ANGII production [137,138]. ANGII enhances hypercellularity, inflammation, ROS generation, renal mesangial cell proliferation, and apoptosis and alters NO availability [139,140].

**Figure 2:**
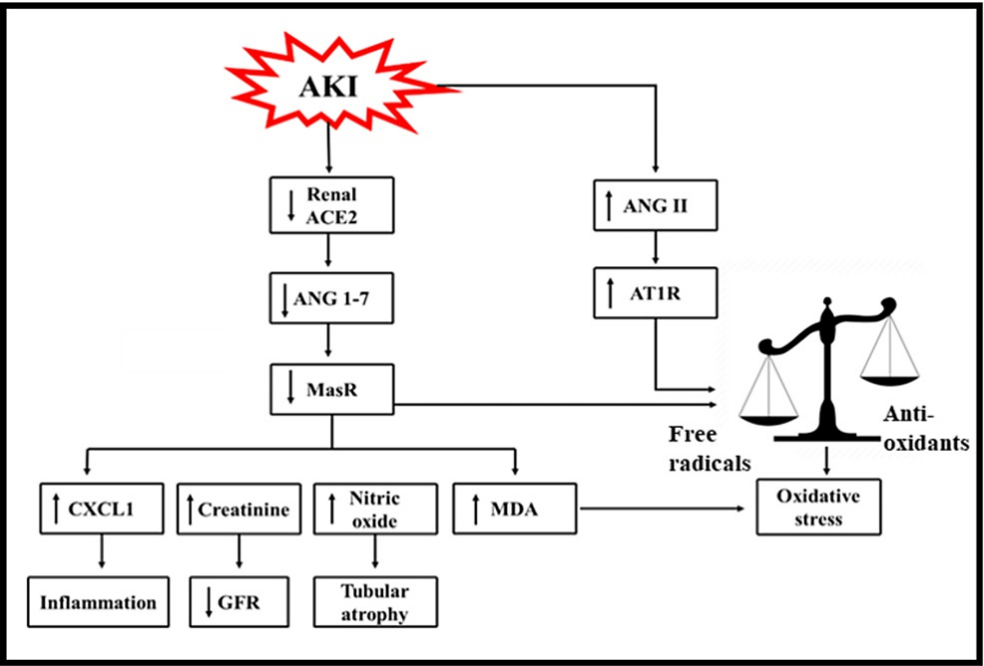
Non-conventional axis (ACE2/ANG1-7/MasR axis) and AKI progression Copyright/license: This figure has been modified from an open-source article [138] distributed under the terms and conditions of the Attribution-NonCommercial-NoDerivs 4.0 International license (CC BY-NC-ND 4.0 DEED). ACE2: angiotensin converting enzyme 2; AKI: acute kidney injury; ANG1–7: angiotensin (1–7); ANGII: angiotensin II; AT1R: angiotensin II type 1 receptor; CXCL1: chemokine (C-X-C motif) ligand 1; GFR: glomerular filtration rate; MasR: Mas receptor; MDA: malondialdehyde.

One of the negative regulators of RAAS is an angiotensin-converting enzyme 2 (ACE2), which converts ANGII to angiotensin (1-7) (ANG1-7) [138]. It is abundantly expressed in glomerular epithelial cells, tubular epithelial cells, and renal vasculature [141]. It has been claimed that ANG1-7 acts via Mas receptor (MasR) and AT2R [142-144]. Acute renal damage alters ACE2 activity. The etiology involves endotoxemia, abnormal hemodynamics, increased NO production, and renal vasoconstriction as detected by two-photon imaging and shown by elevated renal ANGII levels but did not affect ANG1-7. AGT and ACE protein and mRNA levels were elevated, whereas ACE2 activity and expression were decreased [145-149]. AKI decreases ANG1-7, ACE2, and MasR expressions, which increases free radicals, OS, creatinine, nitric oxide, and malondialdehyde (MDA) levels (Figure 2) [138].

Toll-like receptors

One of the most studied pattern recognition receptors (PRR) families is Toll-Like Receptors (TLRs). In animals, there are 13 members, while the human genome has ten members (i.e., TLR-1-TLR-10) [150-152].

Toll-Like Receptors Structure and Characterization

TLRs are interleukin-1 receptor (IL-1R) superfamily type I membrane-associated glycoproteins [153]. TLRs have an extracellular domain (ECD) with 20-27 leucine-rich repeats (LRR) and an intracellular Toll/Interleukin-1 receptor (TIR) domain [154-156]. The cell's TIR domain, analogous to the IL-1R, transmits signals via a complex signaling cascade [157-159]. ECD of TLRs include glycan moieties that bind to ligands [160]. Glycan recognition mechanisms are poorly known [161]. ligand selectivity, signal transduction pathways, and subcellular localization distinguish TLRs [162].

Members of Toll-Like Receptors Family

TLRs are either located on plasma membrane phospholipids of immune cell like TLR-1, 2, 4, 5, 6, and 10 or on lysosomal and endosomal phospholipids where their ligand-binding sites and ECD protrude into the organelles like TLR 3, 7, 8, 9 [150]. TLRs are divided into six families: TLR1, TLR3, TLR-4, TLR5, TLR7, and TLR11 [163]. The TLR1 family includes TLR1, TLR2, TLR6, and TLR10. They are plasma membranes. They form a heterodimeric receptor with TLR2 coupled with a TLR1 family member. TLR-4 and TLR5 localize to plasma membranes [164]. Members of the TLR3, and TLR7 families, on the other hand, are intracellular TLRs expressed in endosomes and lysosomes. They are first localized in the endoplasmic reticulum after synthesizing [165]. The TLR7 family includes TLR7, TLR8, and TLR9 [164]. The first mammalian TLR is TLR-4 [166-168]. TLR members, dimerization, cellular location, ligands, triggered signaling pathway, and product are depicted [150,169].

Expression of toll-like receptors

TLRs are expressed on innate immune cells like macrophages, dendritic cells (DCs), natural killer cells, and circulating leukocytes, including neutrophils and monocytes, adaptive immune cells like T cells and B cells, and non-immune cells like epithelial, endothelial, and fibroblasts [151,170]. TLR mRNA is expressed on non-immune cells in the thymus, tonsils, lymphatic arteries, lymph nodes, spleen, heart, liver, pancreas, colon, small intestine, lung, kidney, ovary, placenta, testis, prostate, skeletal muscle, brain [171].

The kidney's thick ascending limb contains TLR-4 [172]. TLR type-4 is highly expressed in renal cells such as podocytes, mesangial cells, tubular epithelial cells, and endothelial cells [22]. Immunohistochemistry of renal samples showed elevated kidney tubular epithelium TLR-4 expression in urinary tract surgery patients [173].

Toll-Like Receptors and Their Ligand Recognition

TLRs activate the innate immune response and protect hosts against microbial infections [174]. Microbes produce many pathogen-associated molecular patterns (PAMPs), including proteins (e.g., flagellin from bacterial flagella), lipoteichoic acid and peptidoglycan from Gram-positive bacteria, Lipopolysaccharide (LPS) from Gram-negative bacteria, lipopeptides, lipoglycans, lipoarabinomannan, and lipomannans from mycobacteria, zymosan from yeast, double-stranded RNA of viruses, and DNA from viruses [175-177]. Mammalian macrophages and dendritic cells have PRRs on their surfaces or in their cytoplasm to recognize and kill infections [178-181]. PAMPs trigger PRRs, which stimulate the immune system, and endogenous substances are damage-associated molecular patterns (DAMPs) [178-180]. Injury and non-physiological cell death produce endogenous ligands or host-derived DAMPs, such as extracellular matrix components (e.g., hyaluronan and fibrinogen), nuclear and cytosolic proteins (e.g., high-mobility group box protein 1 (HMGB1) and heat shock proteins (HSPs)), and damaged/fragmented organelles (e.g., mitochondrial DNA (mtDNA)) [182,183].

Signaling Pathways of Toll-Like Receptors

Ligands binding to TLR activate intracellular downstream signaling cascades that begin host defensive mechanisms [184]. PAMP-PRR interactions produce pro-inflammatory cytokines and type 1 interferon that targets various microbes [175]. TLR signaling relies on the stimulus, activated TLR, and downstream adaptor molecule. TLR signaling involves two pathways [185].

Myeloid differentiation primary response protein 88-dependent pathway: All TLRs except TLR-3 use it, increasing pro-inflammatory cytokine gene expression [186]. Myeloid differentiation primary response protein 88 (MyD88) recruitment to the activated C-terminal region of TLRs is aided by two key adaptor molecules, MyD88 adaptor-like (MAL) and TIR domain-containing adapter protein (TIRAP). All TLR receptors recruit MyD88 via mal adaptor protein. However, TLR-1, 2, 4, and 6 need an additional association with the TIRAP molecule to facilitate contact with MyD88 [187].

TIR-domain-containing adaptor protein that induces IFN-γ -dependent pathway: It activates interferon type-1 gene expression via TLR-3 and TLR-4. In response to viral dsRNA binding, TLR-3 recruits a TIR-domain-containing adaptor protein that induces IFN- γ (TRIF) directly, but TLR-4 needs another adaptor protein, TRIF-related adaptor molecules (TRAM), to activate TRIF [164,188].

Role of Toll-Like Receptors and Inflammation in AKI

In sepsis, infection prompts a host response in which inflammatory processes help remove infection and repair tissue but can cause organ injury [189]. TLRs' recognition of DAMPs and PAMPs causes leukocyte, endothelial, and epithelial cell activation, which increases adhesion molecule expression, releases more pro-inflammatory mediators and ROS, activates and aggregates platelets, disrupts microvascular function, causes hypoxia, and damages tissue. More DAMPs are produced due to inflammation and tissue damage, which amplifies the inflammatory cascade and results in more tissue damage [84,108,189,190]. After ischemia-reperfusion damage, immune cells from innate and adaptive immune systems contribute to kidney injury and healing [191]. Monocytes and neutrophils mediate the acute phase within 24 h of damage, while T and B lymphocytes mediate renal injury evolution [191,192].

The kidneys may aid immune system homeostasis [193]. Pro-inflammatory cytokines, DAMPs, and PAMPs are filtered in the glomerulus before entering the proximal tubules, where they can directly activate tubular epithelial cells and alter their metabolic and functional status. Inflammatory cells raise tubular and interstitial pressure, generating microcirculatory disruption with hypoxia, tubular blockage from cellular debris, and back-leak. The reduced pressure gradient across the glomerular capillary and tubular space reduces GFR [108].

Complications of AKI

Many factors may connect AKI and death. In heart failure patients, declining GFR and renin-angiotensin system activation produce fluid retention, which causes third-space effusions, peripheral edema, and pulmonary congestion [194]. Lung impairment is a major systemic effect of AKI. AKI may cause lung damage and inflammation, while lung injury causes metabolic and biochemical abnormalities that worsen renal impairment [195]. Furthermore, hyperkalemia is a typical consequence of severe AKI that may cause arrhythmias since potassium excretion is determined by urine output [196]. In addition, acid-base homeostasis is disrupted by AKI. In individuals with AKI, a decrease in their ability to excrete fixed acids results in tubular metabolic acidosis and respiratory compensation in the form of an increased ventilatory drive [197].

Renal replacement therapy (RRT) is necessary because of mortality rates associated with stage 3 AKI, which range from 44% to 52% [198,199]. Observational research found that AKI increases CKD risk [11]. In a two-year cohort analysis of hospitalized Medicare patients, AKI has been linked to a 13-fold higher risk of ESRD in those without CKD and a 40-fold increase in those with both AKI and CKD [3]. AKI increases cardiovascular mortality, and heart failure [200,201].

Current management of AKI

Based on the KDIGO recommendations and bundles of care, the management of AKI is essentially supportive, with the objectives of minimizing additional harm and facilitating the recovery of renal function [38]. Rapid underlying cause identification and treatment are essential. A treatment plan for acute renal damage has been suggested based on an expert opinion [77,202]. Hemodynamic stabilization, early detection of AKI consequences, determining its etiology, and treating it should be the first steps in the therapeutic strategy [203,204]. In renal toxicity, special attention should be paid to drugs to prevent underdosing or adverse effects, which should be stopped, and dose modification based on renal function [203,205]. Further, early antibiotic administration in septic patients is essential [206]. In the treatment strategy for the patient with AKI, it is critical to quickly diagnose and treat associated problems such as metabolic acidosis, hyperkalemia, anemia, and fluid overload. It is also advised to start stress-ulcer prophylaxis and avoid infection during AKI [207].

The appropriate time to start RRT has been a source of debate for many years. KDIGO released guidelines on when to provide RRT to patients who have AKI. Traditionally, RRT is initiated for the acute management of life-threatening complications of AKI, including the following: severe or refractory hyperkalemia (greater than 6 meq/L linked with ECG changes) that is unresponsive to aggressive medical management, or rapidly rising potassium levels, severe or refractory metabolic acidosis (pH less than 7.15) despite medical management, Fluid overload refractory to diuretic therapy, severe azotemia, or clinical complications of uremia like encephalopathy, pericarditis, or neuropathy or certain alcohol and drug intoxications amenable to extracorporeal therapy. Table 2 provides an overview of the relative and absolute recommendations for dialysis [207,208].

**Table 2:** Absolute and Relative Indications for Initiating RRT in Patients With AKI AKI = Acute kidney injury; CKD = chronic kidney disease; RRT = renal replacement therapy. The table is reproduced from [208]

Absolute indications	Refractory hyperkalemia (K^+^ > 6.5 mEq/L, rapidly increasing, or linked with cardiac toxicity)
	Refractory metabolic acidosis (pH ≤7.2 despite normal or low arterial pCO_2_)
	Signs and symptoms of uremia or its complications (bleeding, pericarditis, encephalopathy)
	Refractory pulmonary edema due to fluid overload unresponsive to diuretic therapy
	Toxicity or overdose of easily dialyzable medications or drugs
Relative indications	Limited physiological reserve to tolerate the consequences of AKI
	Severity of the underlying disease
	Advanced nonrenal organ dysfunction worsened or exacerbated by excessive fluid accumulation (i.e., impaired respiratory function)
	Need for large volume fluid administration (i.e., nutritional support, medications, or blood products)
	Concomitant accumulation of poisons or toxic drugs that can be removed by RRT (e.g., salicylates, ethylene glycol, methanol, metformin)
	Anticipating worsening electrolyte problems with AKI (tumor lysis syndrome, rhabdomyolysis)

If there is no absolute indication, evaluate the larger clinical context, including the existence of diseases that can be treated with RRT. It suggests improving resuscitation and ongoing evaluation of the following: the severity and trend of AKI, the severity and trajectory of the disease, and lastly the response to resuscitation optimisation (e.g., intravascular volume, cardiac output, mean arterial blood pressure). Consideration of RRT has been advised if the patient advances to AKI Stage 3. Consider starting RRT if any of the following circumstances are found, even if the patient is on AKI stages 1 or 2: Severe sepsis, permissive hypercapnia, refractory fluid overload and/or accumulation, significantly worsening AKI or disease severity, limited likelihood of early renal recovery, and hypercatabolic condition [209].

**Figure 3:**
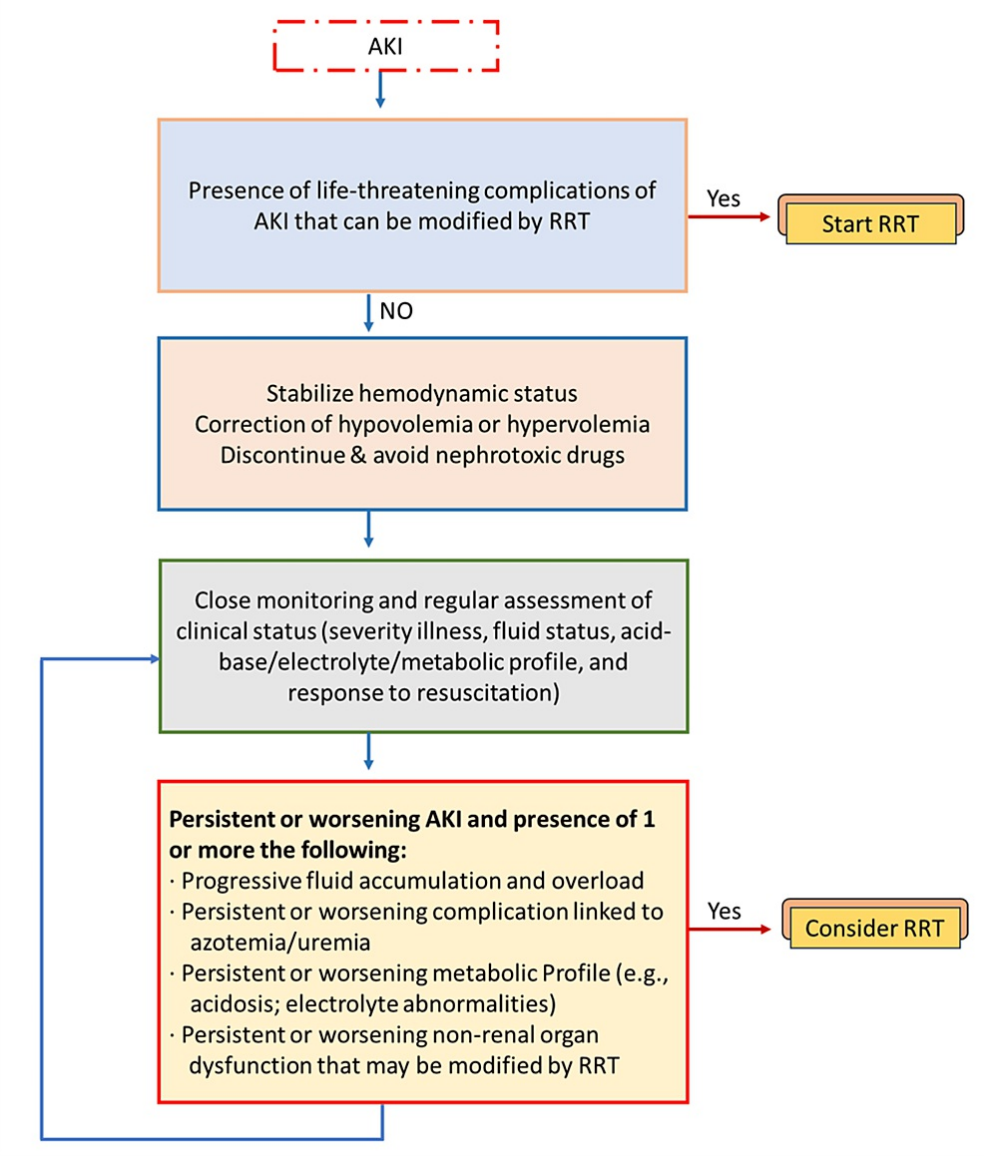
Proposed method for initiating renal replacement therapy (RRT) in critically ill patients with acute kidney injury (AKI). Copyright/license: This figure has been modified from an open-source article [208] distributed under the terms and conditions of the Creative Commons CC BY license.

Also, while deciding whether to begin RRT, take into account the patterns of laboratory test results rather than just the creatinine threshold or BUN [210]. There is debate on the appropriateness of using urea, creatinine, and fluid status/urine output as markers to start RRT in patients with AKI. Serum urea and creatinine, in particular, are not reliable indicators of kidney injury because they take 48 to 72 hours to build up and can be influenced by nonrenal factors like muscle mass, rhabdomyolysis, gastrointestinal bleeding, and medications like corticosteroids (Figure 3) [211].

The rationale for early starting includes the avoidance of severe electrolyte and acid-base imbalances, improved volume control, and the prevention of uremic complications and rapid mortality from the unfavorable consequences of renal failure. Some practitioners begin RRT with the notion that it prevents kidney impairment from becoming worse and helps patients recover from injuries to distant organs caused by fluid overload and electrolyte-metabolic imbalance. This is because early AKI may have a significant influence on other essential organs. RRT may reduce inflammation, which is crucial for those suffering from septic shock [207,212]. When RRT is started before it is truly needed, patients run the risk of a number of negative outcomes, including infections and catheter-related problems, fast fluctuations in electrolyte levels, medication clearance, underdosing of antibiotics, nutrient depletion, hypotension that delays recovery, and a large financial burden [213].

Therapeutic role of toll-like receptor-4 inhibitors

Sepsis-related AKI in critically ill patients has high morbidity and death rates [214]. Understanding AKI pathogenesis lead to novel treatments [215]. Sepsis is caused by severe infections that may lead to systemic inflammatory response syndrome and multiple organ failure [216]. Systemic inflammatory response syndrome is an amplified immune response to unpleasant stimuli, including infection, acute inflammation, reperfusion or ischemia, that localizes and removes the stimulus [217]. The innate immune system releases TNF-α, IL-6, and IL-1β after host-microbe association. "Cytokine storm" [218]. Hence, managing several mediators may be more effective than managing one. Sepsis patients have not reacted to an anti-TNF-α monoclonal antibody or IL-1β receptor antagonist. Nevertheless, other inflammatory signaling pathways are not yet under control [219,220]. This paradigm change emphasizes the importance of the inflammatory response in SI-AKI and firmly implicates TLR-4 as a mediator [221].

Hence, detailed knowledge of TLR-produced signaling pathways may explain immunological processes during SI-AKI, and targeting TLR adapter molecules may provide a potential therapeutic. TLR-4 signaling pathway targets inflammatory diseases and has clinical uses [222]. Infections need TLR-4 activation, but the body cannot manage too much, especially in the latter stages. Even without TLR-4, other TLRs may create interferons. In small animal models infected with influenza A, dengue, Ebola, and respiratory syncytial viruses, TLR-4 antagonists usually lowered cytokines, chemokines, and disease symptoms [223,224].

Drugs targeting TLR-4 are being tested in phase II and III clinical trials for inflammatory diseases, including nonalcoholic steatohepatitis, rheumatoid arthritis, myelodysplastic syndrome, and insulin sensitivity. Certain TLR-4 inhibitors have considerable anti-inflammatory actions and suppress cytokine generation [225]. This is why nephrology experts are interested in the TLR-4 signaling pathway. TLR-4 inhibitors have been tested in various preclinical AKI models [156]. In experimental models of AKI, natural compounds and synthetic compounds have positive benefits. Despite these promising findings, there are no TLR-4-targeted AKI clinical studies. Based on these findings, TLR-4 inhibition may be a promising treatment for the complicated form of SI-AKI's pathogenic effects. Nevertheless, TLR-4 activation and its pro-inflammatory response are crucial for bacterial sepsis clearance [226].

Several natural compounds and several synthetic compounds had beneficial effects in experimental models of AKI were summarized in Carballo et al., study [226]. Resveratrol, a natural phytoalexin, inhibits TLR-4 expression and NF-κB activation in mice and macrophages with LPS-produced AKI. It reduces macrophage infiltration, TLR-4 activation, and renal inflammation. Prevents endothelial cell permeability. It reduced iNOS expression in macrophages [227]. Also, it enhances tubular epithelial cell damage and renal function. It decreases IL-1β, IL-6 and TNF-α, in serum and renal mRNA. Inhibits renal NF-κB [228].

Sulforaphane (SFN), an organosulfur molecule found in broccoli and cabbage, is anti-inflammatory and anti-cancer. SFN inhibits LPS-induced inflammation and TLR-4 activation [229]. It reduces NF-κB activation, TNF-α, and inflammatory infiltration [230].

Paclitaxel, a natural anticancer agent, stabilizes microtubules to prevent their disintegration during cell division or mitosis, causing cancer cells to arrest and die [231,232]. One study demonstrates that paclitaxel may modify non-malignant tissue inflammation and injury. Paclitaxel inhibits NF-kB activation and cytokine production by directly binding MD-2 to prevent MD-2/TLR-4 association. It improves survival rate, decreases IL-1β, IL-6 and TNF-α, and inhibits NF-κB [233].

Curcumin, a polyphenol from Curcuma longa, inhibits IKKβ kinase activity in the MyD88-dependent pathway to reduce NF-κB activation by diverse pro-inflammatory stimuli. Curcumin also decreased LPS-produced IRF3 activation. These findings suggest that curcumin suppresses MyD88- and TRIF-dependent LPS-induced TLR-4 signaling [234]. It reduces SCr and BUN, reduces pro-inflammatory IL-6 and TNF-α and diminishes NF-κB signaling [235]. It reduces kidney damage and inflammatory chemokines, intracellular ROS, neutrophil infiltration, and cellular death. In addition, it reduces TLR-4, TNF-α, NF-κB, and MAPK signaling [236].

Several synthetic compounds like hydrogen sulfide and Eritoran had positive effects in AKI experiment. For a long time, hydrogen sulfide (H2S) was thought to be toxic until endogenous H2S was discovered in the rat brain [237]. H2S is mostly generated from "L-cysteine by two pyridoxal-5′-phosphate-dependent enzymes, cystathionine β-synthase and cystathionine γ-lyase", and one phosphate-independent enzyme, 3-mercapto pyruvate sulfurtransferase (3-MST) [238]. Recently, 3-MST and D-amino acid oxidase uncovered a novel H2S-synthesis route from D-cysteine, which was shown to be superior to L-cysteine in the kidney and cerebellum [239]. It improves renal function, kidney histopathology, OS, inflammation, and TLR-4 expression [240].

Eritoran (E5564) blocks TLR-4 dimerization [241]. It is a synthetic lipid analog and a powerful and selective LPS antagonist. It prevents TLR-4 activation by competitively binding to TLR-4 /MD-2 and preventing LPS from triggering an inflammatory response [242-244]. It reduces SCr, renal histological damage, monocyte infiltration, and inflammatory markers (TNF-α, and IL-1β) [245].

## Conclusions

Despite ongoing efforts, the death rate associated with AKI has remained high over the past half-century. Along with mortality, AKI is a major contributor to the initiation and establishment of CKD and ESRD. The attention has recently shifted away from renal blood flow and toward organ damage brought on by inflammation as a result of new insights into its pathogenesis. Noteworthy, challenges in the search for efficient pharmacological therapy for AKI have arisen due to the multifaceted origin and complexity of the clinical history of people with the disease.
